# The development of an instrument to measure interprofessional collaboration competency for primary care teams in the district health system of health region 2, Thailand

**DOI:** 10.1186/s12875-023-02013-9

**Published:** 2023-02-27

**Authors:** Raphiphaet Prasitanarapun, Nithra Kitreerawutiwong

**Affiliations:** 1 Faculty of Public Health, Naresuan University, Muang District, Phitsanulok Province Thailand; 2 Boromarajonani College of Nursing, Uttaradit, Faculty of Nursing, Praboromarajchanok Institute, Ministry of Public Health, Nonthaburi, Thailand

**Keywords:** Interprofessional collaboration, Competency, Instrument development, Primary care

## Abstract

**Background:**

Evidence shows that interprofessional collaboration (IPC) practice contributes to the quality of health care. However, there are limited instruments to assess IPC in providing primary care in the district health system (DHS) in Thailand. The aim of this study is to develop a valid and reliable instrument to assess the IPC competency of primary care team members in DHSs.

**Methods:**

This study was designed as an exploratory mixed methods study. In the qualitative phase, 37 participants, including policymakers, practitioners, and academics with experience in primary care, were involved. Data were analysed using thematic analysis, and trustworthiness was verified by triangulation and peer debriefing. In the quantitative phase, content validity, exploratory factor analysis (EFA), confirmatory factor analysis (CFA), and reliability were conducted, and the final version of the questionnaire was evaluated with 497 participants.

**Results:** The findings showed an I-CVI range of 0.86–1.00 and S-CVI/UA = 0.87 for 49 items with a 5-point Likert scale. EFA suggested six factors: 1) collaborative teamwork, 2) population- and community-centred care, 3) communication and mutual respect, 4) clarification of roles and responsibilities, 5) interprofessional reflection, and 6) interprofessional values and mixed skills. In the CFA results, the model fit indices were acceptable (CFI = 0.99, RMSEA = 0.049, SRMR = 0.043) or slightly less than the goodness-of-fit values (GFI = 0.84). All subscales showed acceptable Cronbach’s alpha values with a range of 0.86–0.94.

**Conclusions:**

The developed IPC competency instrument was confirmed its validity and reliability that contributes to assessing the IPC competency of primary care teams in DHSs. This information provides evidence to support tailored intervention to promote the IPC competency of primary care team work to achieve a common goal.

## Background

Interprofessional collaboration (IPC) has been documented as a vital component in research, education, and health care practice [1, 2]. The World Health Organization [3] defines IPC as “collaborative practice that happens when multiple health workers from different professional backgrounds work together with patients, families, carers and communities to deliver the highest quality of care across settings”. The World Health Professions Alliance [4] described the benefits of IPC as improved access to health interventions and improved coordination between different sectors for individuals and their families with more involvement in decision-making, providing comprehensive, coordinated care, the efficient use of resources, the reduced incidence and prevalence of disability, and increased job satisfaction of health professionals. Previous studies have suggested improving IPC when providing holistic care for older adults [5], enhancing collaborative management to achieve optimal care for people in the district health system [6], providing appropriate chronic condition management in primary care [7], delivering patient-centred care and improving patient and system outcomes [8]. Leading IPC requires training in new knowledge and skills [9].

According to the concept of IPC defined by the World Health Organization (WHO), which involves all sectors’ engagement in health and emphasizes patient-centred care [10], differences in the context of the implementation of IPC in hospitals, primary care facilities, and educational institutions are influenced by various factors, including sociocultural characteristics within an institution or within each group of health profession teams [11]. In Thailand, primary care services provided through a network that called district health system (DHS), provide health promotion, disease surveillance, home healthcare, out-patient services with supervision and support by medical doctors and health care provider from district hospitals for Thai citizen under universal health coverage. The health care provider called primary care team that are composed of various professions, including physicians, nurses, public health professionals, Thai traditional medicine practitioners, and allied health providers working in facilities and community settings. In addition, community health funds and community-based long-term care schemes are provided in the DHS with the collaborative work of the health sector, local sector, and community sector [12, 13], which involve challenges in implementing them across the settings of people’s homes, communities, and facilities*.* Barriers to IPC in the primary care setting include team characteristics and team processes, such as role clarification, communication, a lack of formal team structure and leadership, limitation of co-location of services, and the absence of commitment goals [6, 14–17]. Therefore, an instrument for the assessment of IPC competency is needed to identify the gap in the development of collaborative competencies among primary health care teams.

A scoping review of IPC competency instruments in health care reported that different instruments are used in various health care settings, such as hospital settings, and with different populations, such as social workers, nurses, and physicians, depending on the measurement purpose [18]. Instruments for measuring general IPC competency include the Interprofessional Collaborative Competency Attainment Survey (ICCAS) [19], the Chiba Interprofessional Competency Scale (CICS29) [20], the Collaboration S﻿cale between C﻿ommunity Nurses (CNs) and General Practitioners (GPs) in primary health care teams (COPAN scale) [21], and the Collaborative Practice Assessment Tool (CPAT) [11]. These instruments may be applicable in diverse health systems and cultures. Due to the specific Thai health care system that emphasizes working across sectoral approaches to primary health care at the district level [22], an accurate evaluation instrument is needed. Regarding IPC competencies in particular, limited study of the IPC instrument in primary care in Thailand has been conducted. As such, this study aims to develop and validate an IPC competencies instrument to evaluate interprofessional skills for collaboration in primary care to provide an effective instrument for assessing and monitoring the IPC competency needed by all members of the primary care team.

## Methods

### Study site

This study was conducted in health region 2, covering 5 provinces, including Phitsanulok, Petchboon, Sukhothai, Tak, and Uttaradit. This area was purposively selected because it is a medical hub in the lower northern region, contains a good mix of several types of primary care facilities, and covers the cultural diversity of the population.

### Study design

**Fig. 1:**
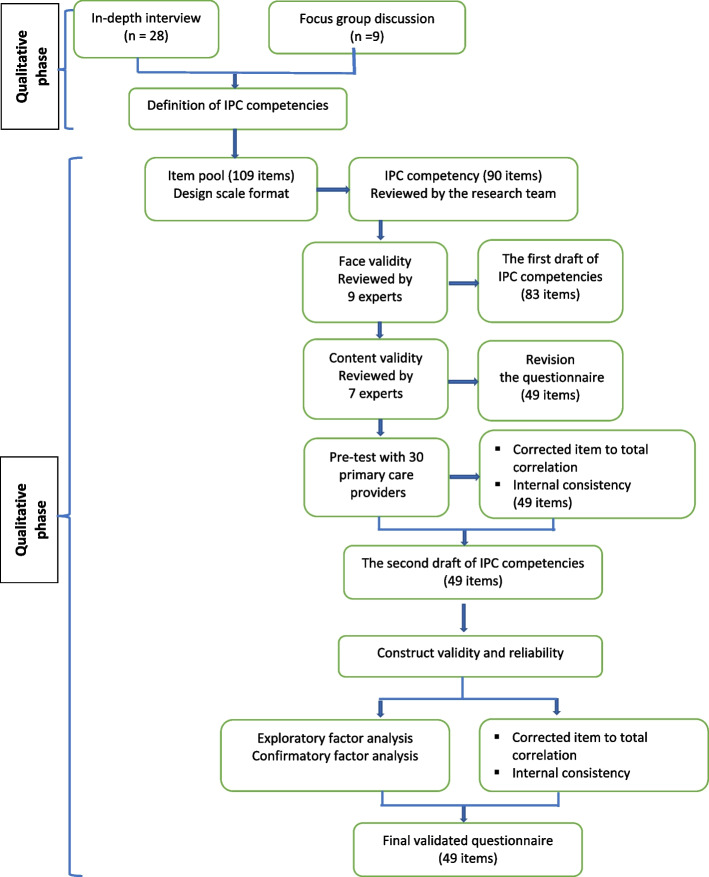
Sequential exploratory mixed methods design in the development of Interprofessional Competency (IPC)

This study employed a mixed-method, sequential exploratory design for the development and validation of the IPC competency instrument. The exploratory design began with a qualitative data collection and analysis phase, which developed to a subsequent quantitative phase. The displays demonstrated the potential to connect data of the qualitative findings to items of the instrument. Then, an instrument development joint display mapped the qualitative dimensions of IPC competency to quantitative instrument items [23]. In the qualitative phase, an in-depth interviews and focus group discussions to understand the concept of IPC competencies was conducted. The item pool was established based on the known components and findings from the qualitative results. In addition, the items from the literature that relevant to the definition of qualitative results were employed as the item pool. In the quantitative phase, the item pool was tested for validity and reliability. The mixed method, sequential exploratory design was conducted in the following steps: 1) a qualitative phase to assess the concepts and components of IPC competencies and 2) a quantitative phase to test the questionnaire validity and reliability. Figure 1 presents the process of developing an instrument for assessing the IPC competencies of primary care teams.

### Phase 1: qualitative phase to assess the concepts and components of IPC competencies

#### Qualitative study

The qualitative approach was conducted with in-depth interviews and focus group discussions (FGDs) to explore the concept of IPC competencies in the Thai DHS. In-depth interviews were employed to explore new issues and provide detailed information on 28 policymakers, academics, and practitioners. The FGD was designed to encourage discussion between the 9 practitioners. This study included 2 groups with four and five patients in each group. Purposive sampling was used to achieve a wide range of perspectives [24] from different disciplines. The inclusion criteria for this study were as follows: 1) at least 2 years of experience in primary care policymaking for human resource development in primary care at the national, regional, and provincial levels for each of 2 participants at each level, 2) published research in the field of human resource development in primary care within 5 years for 2 participants, and 3) health care providers who worked within interprofessional teams for at least 2 years. Primary care professionals were included from disciplines representing family physicians, dentists, registered nurses, physical therapists, Thai traditional medicine practitioners, public health practitioners, psychologists, and radio therapists. The participant from each discipline was recruited 3–4 persons to invite in participating in in-depth interview.

Informed consent was obtained before data collection with voluntary participation. Semi-structured interview questionnaires were used. The key questions were 1) In your opinion, what are the important of the attribute of primary care team who worked in district health system (attribute refer to knowledge, attitudes, and trait)?, 2) What is the definition or main concept of interprofessional collaboration?, 3) Please share your experiences regarding interprofessional collaboration of primary care team in DHS., 4) What is your ideology interprofessional collaboration of primary care team in DHS?, and 5) What are the room of improvements of interprofessional collaboration of primary care team in DHS?. Each question was an open end and can be probed when the issue emerged in the field work of data collection. The time lasted between 40 and 60 min for in-depth interviews and 72–90 min for focus group discussions [25]. Interviews were recorded and transcribed verbatim. Data were collected until saturation was reached at which no new data emerge in data collection.

The process to enhance trustworthiness was direct verbatim quotes to support the findings, and personal data triangulation and within-method data triangulation were conducted to increase credibility [26]. Peer debriefing by reviewing and assessing transcripts, identifying emerging and final categories from the transcripts, and collecting the final findings of themes and definitions of IPC competencies were employed to enhance credibility [27]. The data analysis was conducted independently by the two authors and discussed until consensus was reached. Data were analysed using inductive thematic analysis that involves reading through verbatim transcript data and identifying and coding emergent themes within the data. Data were independently code by the two authors to obtain the key themes and definition of IPC competencies. Each two authors analysed the similar transcript. All coding and interpretation were discussed by the researchers until consensus reached. This step obtained the components and definition of IPC competencies.

### Phase 2: the quantitative phase to test the questionnaire’s validity and reliability

#### Item pool and design the instrument format

The item pool was established based on the qualitative results and a literature review. The format of measurement was determined. Likert scales were selected to measure psychological constructs because they do not require a simple yes/no answer from the respondent but rather allow for degrees of opinion or even no opinion at all [20, 21, 28–30]. The instrument used a five-point Likert scale: 1 = lowest performing, 2 = low performing, 3 = moderate performing, 4 = high performing, and 5 = highest performing. Higher scores reflect high competence. However, social desirability bias may occur if the respondents choose answers based on what they think is socially acceptable [31]. Anonymizing respondents, ensuring confidentiality, and using neutral and nonsuggestive question wording were applied to mitigate social desirability bias [32]. The first author pooled the items and obtained 109 items. The second author reviewed, and the redundant items were reduced, yielding 90 items.

#### Face validity

To assess whether the items of each domain were sensible, appropriate, replicated, and relevant to the respondents [33], nine experts from different fields, including researchers in human resource management and instrument development, physicians, dentists, pharmacists, nurses, physiotherapists, Thai traditional medicine practitioners, and public health practitioners, were invited to complete the questionnaire. The experts reviewed and eliminated 8 items, leaving 83 items.

#### Content validity

For content validity, 7 experts in the fields of primary care (2 people) and instrument development (2 people), physicians (1 people), nurses (1 people), and public health practitioners (1 people) examined the first draft of the questionnaire. The experts were not the same group as in the face validity assessment to obtain various perspectives on validity. They considered the domains and definitions, relevance and clarity of items, linguistics (e.g., terminology, simplicity), and the adequacy and appropriateness of item response of the instrument [34]. The item-level content validity index (I-CVI) considered on the agreement of the experts. A panel of content experts is invited to rate each scale item in terms of its relevance to the underlying construct. For the scale-level content validity index through the universal agreement (S-CVI/UA) was defined as the CVI for the whole instrument. The I-CVI scores and a S-CVI/UA score were calculated. Values of I-CVI and S-CVI/UA ≥0.80 were recommended [35, 36]. The I-CVI range was 0.86–1.00, and S-CVI/UA = 0.87 was acceptable. After this step, 49 items remained.

#### Construct validity

Construct validity testing was incorporated as part of the IPC competency development. For construct validity, the sample was multiplied by 12 (12 times*50 items that were rounded up from 49 items of the second draft questionnaire = 600). This number was greater than 500, which is suggested for very good for factor analysis [37]. The sample included 600 primary care practitioners who worked in health region 2 with stratified random sampling. Data analysis for construct validity included two phases.

##### Exploratory Factor Analysis (EFA)

EFA can identify items in the instrument that reflect the dimension of IPC competencies [38]. Varimax rotation, orthogonal rotation and principal component analysis extraction was used to explore the existing factorial pattern.

##### Confirmatory Factor Analysis (CFA)

CFA was used to evaluate the internal structure of the IPC competencies. It confirmed the hypothesized number of constructs, the relationship between the constructs, and the relationship between the constructs and the items [39].

#### Reliability

Internal consistency was calculated as Cronbach’s alpha for each domain. The value of the corrected item to total correlation and the alpha if an item was deleted were also evaluated. Cronbach’s alpha values ranging from 0.70 to 0.95 and corrected item total correlations greater than 0.20 were acceptable [34, 40]. The step was carried out after assessing the content validity index and confirmatory factor analysis.

### Statistical analysis

EFA was used to examine the factor structure in the instrument. Kaiser–Meyer–Olkin (KMO) and Bartlett’s test of sphericity were performed to evaluate the adequacy of the sample size and the correlation between the extracted factors. The Scree test was used to determine the number of factors to retain. Then, the Orthogonal varimax rotation was used to clarify the relationship among factors. This technique minimizes the number of variables that have high loadings on each factor and simplifies the interpretation of the factors. Three criteria used in retaining items and determining the factors were 1) factor loading ≥0.30 [41], 2) no cross-loading with a difference below 0.2 at each step of iteration [42], and 3) each factor should have at least three items [43]. The questionnaire retained 49 items within six dimensions, and the internal consistency was confirmed. Confirmatory Factor Analysis (CFA) was performed for both the first- and second-order CFA models. The indices used to examine the goodness-of-fit of the model were considered as the Chi-square per degrees of freedom (χ2/df) ratio (< 5), 2) Comparative Fit Index (CFI > 0.90), 3) Root Mean Square Error of Approximation (RMSEA< 0.08), 4) Goodness-of-fit Index (GFI ≥ 0.90), and 5) Standardized Root Mean Squared Residual (SRMR < 0.08) [44–46]. Finally, the internal consistency in each factor was examined by Cronbach’s alpha.

### Ethics approval

This study was approved by the Naresuan University Research Ethics Committee (Code no P3–00027/2563). Informed written consent was obtained from all participants before data were collected. Permission for research access was obtained from the Provincial Medical Office, Community Hospital, and District Health Office of 5 provinces, including Phitsanulok, Petchboon, Sukhothai, Tak, and Uttaradit Provinces.

## Results

### Results from the qualitative study

The inductive analysis was conducted to formulate the themes. The results from the analysis of qualitative data included 1) clarification of the role and responsibility, 2) collaborative teamwork, 3) interprofessional value, 4) communication, 5) reflection, and 6) population- and community-centred care (Table 1).

**Table 1:** IPCP competencies and their definitions for primary care teams in the DHS

Theme	Definition
1. Clarification of roles and responsibilities	Awareness of the roles and responsibilities among team members, aligned with shared commitment to the district health system’s goal and responsibility to promote health and prevent diseases in the population.
2. Collaborative teamwork	Working together with other professions and application of individual expertise to build collaborative relationships for effective teamwork in relation to leadership, motivation, management of work procedures, and proactive services to create a positive working environment.
3. Interprofessional value	Recognition of value in the population and respect of interprofessional teams with shared values in working together.
4. Communication	Intra- and inter-team communication to coordinate collaboration among the primary care team members to promote, prevent, cure, rehabilitate, and provide counselling.
5. Reflection	Experience-based learning through reflection and critical appraisals of team members to improve work performance.
6. Population and community-centred care	Working collaboratively with the population, community, and all sectors in a district according to the health needs of the population and community, as well as the monitoring of health outcomes.

### Results from the quantitative study

Demographic characteristics. The response rate was 82.3% [(497/600) * 100]. The attributes of the participants are presented in Table 2. Of the respondents, 73.80% were female, 59.80% were married, and the most common age group was 36–50 years (43.7%) (mean = 47.34, SD. = 7.12). Most of the sample (81.30%) graduated with bachelor’s degrees. A total of 39.4% were public health practitioners, and 76.0% worked in subdistrict health-promoting hospitals. Their experience in service was 2–40 years (mean = 17.65, SD. = 10.40), and the experience of working in primary care ranged from 2 to 39 years (mean = 17.65, SD. = 10.40).

**Table 2:** Demographic characteristics of the respondents (*n* = 497)

Variable	Attribute	Number	Percent
Sex	Male	130	26.2
	Female	367	73.8
Marital status	Single	161	32.4
	Married	297	59.8
	Widowed/Divorced/Separate	39	7.8
Age (years)	< 35	187	37.6
	36–50	217	43.7
	≥ 51	93	18.7
(Mean = 40.58, SD = 9.60)			
Educational level	Bachelor degree	404	81.3
	Master degree	91	18.3
	Doctoral degree	2	0.4
Professions	Physician	27	5.4
	Dentist	25	5.0
	Pharmacist	22	4.4
	Nurse	165	33.2
	Physiotherapist	25	5.0
	Thai Traditional Medicine	37	7.5
	Public Health Practitioner	196	39.5
Type of workplace	Primary care unit	2	0.4
	Hospital	117	23.5
	Sub-district health promotion hospital	378	76.1
Experience in health services (years)	< 10	172	34.8
	11–20	125	25.1
	≥ 21	200	40.1
(Mean = 17.65, SD = 10.40)			
Experience in primary care services (years)	< 10	246	49.5
	11–20	127	25.6
	≥ 21	124	24.9
(Mean = 13.96, SD = 10.01)			

#### Exploratory factor analysis

For IPC competencies, the Kaiser-Meyer-Olkin (KMO) test and Bartlett’s test of sphericity were 0.93, and Bartlett’s test confirmed that factor analysis was appropriate (χ2 = 19,926.28, df = 1176, *p* value < 0.001). Varimax rotation was used to extract the factorial pattern. A total of six factors were extracted and rotated, and the cumulative variance explained was 66.53%, with eigenvalues from 1.02 to 24.34. The factor loadings range from 0.411–0.737. The overall score of Cronbach’s alpha coefficient was 0.97 with a range of 0.86–0.94. The results of the factor analysis are shown in Table 3.

**Table 3:** Factor loading and item statements

Dimensions and items	Factor loading
**Factor 1 Collaborative teamwork** (13 items with eigenvalue = 24.34, % of variance = 49.68, Cronbach’s alpha coefficient = 0.94)	
i15 Taking part in the decision-making for the service with the primary care team.	0.681
i16 Being able to participate in managing the care plan for the population, clients, and community with the team.	0.681
i13 Being able to assist team members in working together productively to achieve positive outcomes for the population, clients, and community.	0.633
i20 Being able to foster a suitable environment throughout the discussion or meeting so that other disciplinary teams can express their opinions.	0.626
i19 When there is conflict within the team, being able to control and resolve conflict with the principles.	0.619
i11 Expressing leadership and followership in managing the support of interprofessional collaboration (such as being responsible for data collection, presentation of the meeting, and sharing roles in collaboration).	0.609
i9 Being able to consistently inspire the team to work collaboratively in interprofessional collaboration.	0.598
i14 Being able to integrate interprofessional knowledge and disciplines to fulfil a team-goal commitment.	0.586
i10 Managing the process in interprofessional collaboration among the primary care team (such as collaborating, managing an effective working system, and planning).	0.581
i12 Being able to foster an environment to create innovation within the team.	0.575
I17 Being able to offer proactive care with the interprofessional team to respond to health needs.	0.570
i18 When discrepancy arises during collaboration, being able to manage and analyse causes and factors to resolve the issue.	0.568
i21 Appropriately applying knowledge and expertise in your own disciplines to work with the team.	0.442
**Factor 2 Population- and community-centred care** (8 items with eigenvalue = 2.38, % of variance = 4.87, Cronbach’s alpha coefficient = 0.93)	
i47 Collaborating with other sectors, such as a representative of people, the private sector, and the local sector, in solving public health problems.	0.737
i48 Working in the area with concern for the individual, population, and community for which you are responsible.	0.730
i45 Providing the individual, family, and population with trustworthy heath information, news, and knowledge so that they can make informed health decisions.	0.710
I49 Providing supportive advice to people and the community with the primary care team and other sectors.	0.707
i46 Obtaining solutions to public health problems for local people in collaboration with the primary care team.	0.698
i42 Creating activities to encourage the population to participate in mutually working as a team for the population, clients, and community.	0.660
i44 Providing channels to receive information about health problems and the recommendations of clients and people for the planning of health care with the primary care team.	0.659
I43 Evaluating the health needs of the population, clients, and community in several dimensions to design a holistic health care service for the primary care team.	0.609
**Factor 3 Communication and mutual respect** (10 items with eigenvalue = 1.83, % of variance = 3.75, Cronbach’s alpha coefficient = 0.92)	
i29 Being open to shifting perspectives when faced with new facts that are useful for interprofessional collaboration.	0.719
I27 Expressing gratitude and respecting the roles and responsibilities of each discipline in the team.	0.689
i28 When discrepancy occurs within the team, being able to respond to one another honourably and creatively.	0.671
i32 Providing two-way communication in the interprofessional collaboration.	0.578
i33 Using information regarding patient, clients, and populations accurately and deliberately for communication within the interprofessional team in providing services.	0.571
i34 Using understandable language, standard language, or accurate medical terms when communicating with the interprofessional team.	0.567
i26 Accepting cultural discrepancies and diversity among the members of the interprofessional team.	0.548
i31 Selecting communication channels and methods, including an information technology system, for effective communication of the interprofessional team.	0.543
i35 Being able to communicate with the relatives, families, and community of the clients to enhance their understanding of the working process of the primary care team.	0.537
i30 Being able to communicate with the interprofessional team regarding tasks that you are responsible for to increase understanding among the team and accurately and effectively transfer task to each other.	0.509
**Factor 4 Clarification of roles and responsibilities** (8 items with eigenvalue = 1.67, % of variance = 3.41, Cronbach’s alpha coefficient = 0.90)	
i2 Appropriately sharing the work and responsibilities of each discipline to achieve the goal of the team.	0.729
i6 Ensuring for accuracy in the work that that you are responsible for according to your discipline area to achieve the goal of the team.	0.691
i5 Presenting information, views, and ideas to care for the population and clients according to your discipline area.	0.671
i3 Following the practice for the job that you are responsible for according to your disciplinary area.	0.669
i1 Following the practice according to the role of the discipline during interprofessional collaboration.	0.631
i4 Creating a collaborative atmosphere by supporting the team members to express opinions according to their skills and expertise of their professions.	0.618
i8 Evaluating the working outcome according to the role of responsibility of the interprofessional team.	0.617
i7 Supporting the sharing and monitoring of the aims and *outcomes for the populations that are served by the team.*	0.573
**Factor 5 Interprofessional reflection** (6 items with eigenvalue = 1.34, % of variance = 2.73, Cronbach’s alpha coefficient = 0.92)	
i38 Explaining the perspectives, feelings, and stress in collaborative work.	0.729
i40 Reviewing and analysing, depending on the situation, circumstances and factors that led to failure during collaborative work.	0.709
i41 Summarizing the learning based on interprofessional collaboration to improve the teamwork.	0.685
i39 Providing feedback during the process of interprofessional collaboration to effectively improve the working process.	0.682
i37 Exchanging information, viewpoints, experiences, and suggestions after finishing the interprofessional collaboration to achieve the goal of providing health care services to the population, clients, and community.	0.673
i36 Regularly reviewing your own roles and the work of the interprofessional team.	0.549
**Factor 6 Interprofessional values and mixed skills** (4 items with eigenvalue = 1.02, % of variance = 2.08, Cronbach’s alpha coefficient = 0.86)	
i25 Discussing the benefits of interdisciplinary collaboration and obtaining opinions from population, clients, and community regarding the advantages of interprofessional work.	0.557
i24 Obtaining feedback from all stakeholders, such as population, clients, and community, to increase client safety, continuity of care, and high-quality patient-centred care.	0.554
i23 Planning projects that intend to increase the team’s appreciation for and pride in the interprofessional teamwork for delivering care to the population, clients, and community.	0.492
i22 Being able to perform the functions of other team members in the DHS as appropriate for the circumstances.	0.411

#### Confirmatory factor analysis

**Fig. 2:**
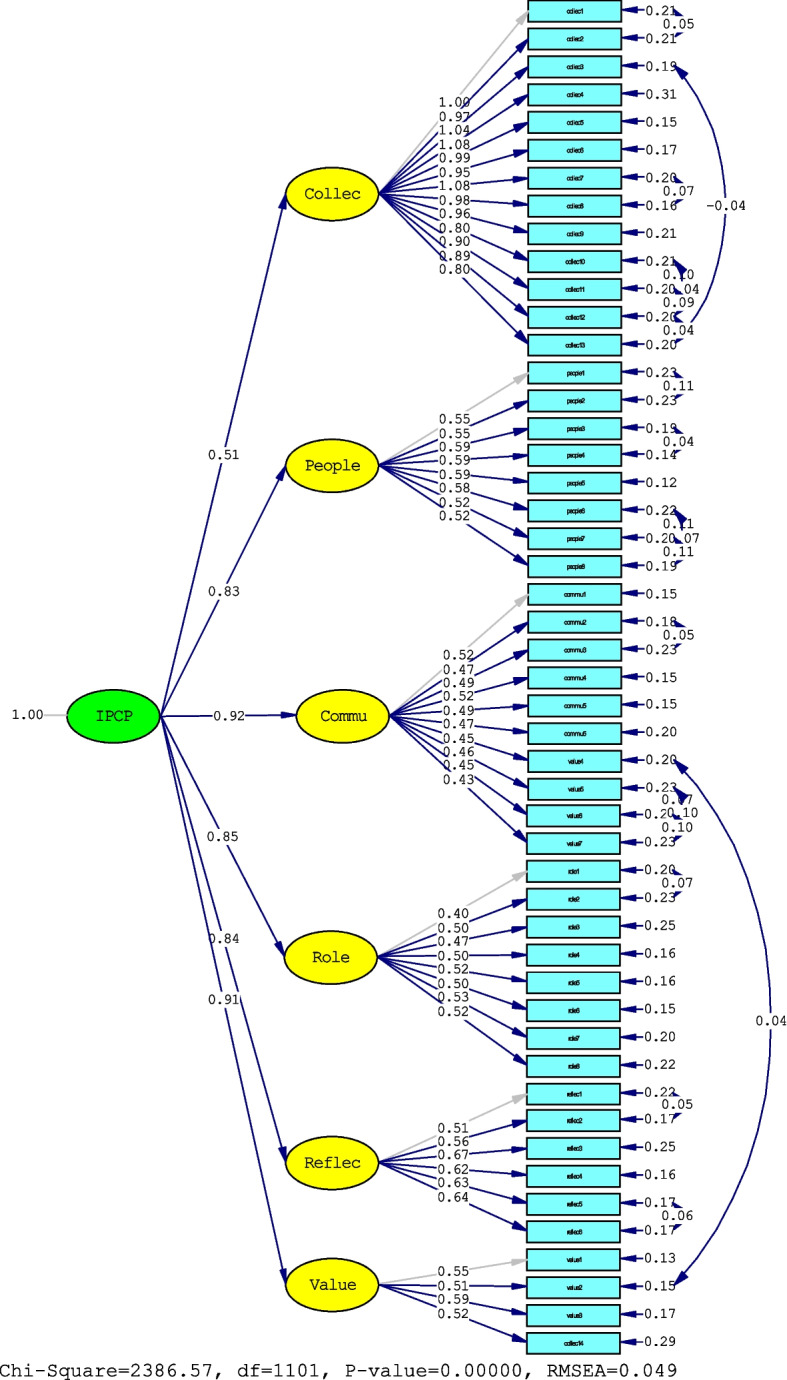
Second order confirmatory factor analysis factor loadings of the IPC competencies of primary care team. Note: Collec = Collaborative teamwork, People = Population- and community-centred care, Commu = Communication and mutual respect, Role = Clarification of roles and responsibilities, Reflec = Interprofessional reflection, and Value = Interprofessional values and mixed skills

The results of CFA showed that the first-order factor loading ranged from 0.43–0.67. All factor loadings were statistically significant (*p* < 0.001). In addition, the standardized factor loading for the second-order factor model is presented in Fig. 2. The results of the model fit indices are reported in Table 4.

**Table 4:** Results of confirmatory factor analysis for the first order and second order model of IPC competencies scale

Model		χ^2^	df	χ^2^/df	CFI	GFI	RMSEA	SRMR
First-order	Pre-modification fit indices	3716.32	1112	3.34	0.98	0.77	0.069	0.043
	Post- modification fit indices	2055.73	1077	1.91	0.99	0.86	0.043	0.039
Second-order	Pre-modification fit indices	3781.61	1121	3.37	0.98	0.76	0.069	0.046
	Post- modification fit indices	2386.57	1101	2.17	0.99	0.84	0.049	0.043

abbreviations were described as follows: IPC competencies scale = Interprofessional competencies scale, *χ*^*2*^ chi square, *df* Degree of freedom, *CFI* Comparative fit index, *GFI* Goodness fit index, *RMSEA* root mean square error of approximation, *SRMR* Standardized root mean squared residual

#### Reliability

Internal consistency reliabilities and Cronbach’s alpha for the subscales are presented in Table 5. Cronbach’s alpha coefficient of the subscale ranged from 0.86–0.94, with an overall score of the instrument of 0.97. According to the analysis results, the alphas if individual items were deleted, Cronbach’s alpha of the corresponding factor increased slightly (Range for the overall score of, the alphas if individual items were 0.94–0.97). Therefore, the extracted factors had good internal consistency.

**Table 5:** Cronbach’s alpha coefficient and its subscales

Dimensions	Number of items	Corrected item-total correlations	Cronbach’s α	Alphas if individual item deleted
1. Collaborative teamwork	13	0.69–.0.80	0.94	0.93–.0.94
2. Population- and community-centred care	8	0.73–.0.81	0.93	0.92–.0.93
3. Communication and mutual respect	10	0.68–.0.72	0.92	0.91–.0.92
4. Clarification of roles and responsibilities	8	0.65–.0.74	0.90	0.89–.0.90
5. Interprofessional reflection	6	0.70–.0.81	0.92	0.90–.0.91
6. Interprofessional values and mixed skills	4	0.61–.0.76	0.86	0.80–.0.86
**Total**	**49**	0.58–0.76	0.97	0.94–0.97

## Discussion

The aim of this study was to develop and validate the IPC competencies instrument to evaluate the interprofessional collaborative competencies of primary care teams in DHSs. The domains and definitions of IPC competencies obtained from qualitative methods from all stakeholder coverage at the national, regional, and provincial levels were appropriate for the primary care system in Thailand. A two-stage sequential mixed method in designing an instrument of this study is consistent with the study in health professional from four different hospitals in Japan [47], health, medical, welfare, and education field in Japan [29], and undergraduate student in complementary medicine in Germany [48]. The approach led to understand the working environment of collaborative practice in DHS of the specific context in Thailand. This approach provided the data connection between the qualitative and quantitative phases. Qualitative data were provided for each question in the item pool in the quantitative phase. This approach increases the credibility of qualitative findings that are congruent with quantitative findings [49].

The questionnaire consisted of 49 items in 6 dimensions, including 1) collaborative teamwork, 2) population- and community-centred care, 3) communication and mutual respect, 4) clarification of roles and responsibilities, 5) interprofessional reflection, and 6) interprofessional values and mixed skills. The items had an acceptable factor loading in the range of 0.411–0.737, which explained 66.53% of its variation. The I-CVI range of 0.86–1.00 and S-CVI/UA = 0.87 confirmed acceptable content validity. For the construct validity, all items had a factor loading value > 0.3. The reliability was examined by Cronbach’s alpha coefficient of the subscale range from 0.86–0.94 with the overall score of the instrument of 0.97. The work of Nunnally [50] showed that the lower cut-off (i.e., 0.70) is appropriate in the early stages of research, such as during instrument development. Additionally, Nunnally [50] suggested that reliability coefficients should be used for preliminary research (≥ 0.5–0.6), basic research (≥ 0.8), and applied research (≥ 0.9–0.95), which is consistent with George and Mallery [51], who suggested a tiered approach consisting of the following: “≥ 0.9 = Excellent, ≥ 0.8 = Good, ≥ 0.7 = Acceptable, ≥ 0.6 = Questionable, ≥ 0.5 = Poor, and ≤ 0.5 – Unacceptable”. This study was the first stage applied instrument development of a questionnaire that will be used in applied research, and the obtained subdimension and overall instrument values were acceptable. These results confirmed that all items and construct validity were acceptable. This instrument had acceptable values consistent with previous studies [11, 19–21]. Therefore, this instrument can be used in assessing IPC competencies to confirm and validate the questionnaire in a large sample size.

For discussion of the model fit of CFA, when considering the first model and the second model, the GFI values of this sample were 0.86 and 0.84, respectively. This result can be explained by the fact that the GFI may be affected by external factors such as sample size, the number of parameters, and the degrees of freedom to sample size ratio and does not reflect poor model fit [52]. The CFI value was close to 0.9, which shows a relatively good fit. The other fit indices, CFI, RMSEA, and SRMR, were within the acceptable values for both the first order and second-order models [44–46]. Therefore, the results showed a satisfactory model fit of 6 factors.

The IPC competencies obtained 6 factors (or dimensions), including 1) collaborative teamwork, 2) population- and community-centred care, 3) communication and mutual respect, 4) clarification of roles and responsibilities, 5) interprofessional reflection, and 6) interprofessional values and mixed skills. The two dimensions were clarification of role and responsibility and collaborative teamwork, which were consistent with the previous studies [19–21]. The reason can be explained by each team member’s clear identity role and the interdependence of the team, which promotes successful interprofessional teamwork and improves patient care outcomes [53]. In addition, when working with various disciplines in the primary care team in a DHS, clarifying the roles and responsibilities of each member is critical to team success. However, role clarity is not always easy; some established roles have clear delineation, while other newer or complex roles may have responsibility overlap. Therefore, assessing this domain is critical to improve the knowledge and skills of primary care teams [54].

The dimensions of population- and community-centred care were similar to those in previous studies [11, 19, 20]. According to the health care transition towards patient-centred, community-based care and home care, it is becoming increasingly important to train health care providers to achieve competency in the arena of patient-centred care [10]. Moreover, Thailand provides primary care through DHSs, such as community health funds, long-term care funds with home health care services at the patient’s home, and noncommunicable disease prevention and control [13, 55, 56]. Consequently, the collaboration of professionals at provincial district hospitals, and subdistrict health-promoting hospitals in providing resources and technical support enhances the capacity of primary care teams regarding knowledge and skills in population and community-centred recommendations.

Regarding communication and mutual respect, this dimension was consistent with other studies [19, 57]. This dimension emerged because in the DHS, there are various professions with different perspectives on working together; therefore, communication among team members, clients, and the population is essential for team functioning to transfer accurate information in a timely manner. Consistent with the study of Busari et al. [58], interprofessional communication and key improvement areas seem suitable for small-scale, limited resource settings. Moreover, the sharing of patient information should be the prioritized focus in communication improvement. In addition, an open and effective communication channel among health teams allows professionals to share their anxieties and daily victories, which contributes to improved health results and increased user satisfaction [59]. Mutual respect is relevant to professionals who contribute to others involved in the work process and considering the impact of their own actions on others’ ability to do their work [60]. This dimension is important to the primary care team in the Thai DHS due to the work process based on the vertical and horizontal hierarchy. Communication with respectful listening and mutual respect will contribute to coordination among teams.

Considering the combination of interprofessional value and skill, this dimension was likely meaningful to the study of Jaruseviciene et al. [21], who used the phrase diffusion of functions. The findings can be explained by the fact that in Thailand, emphasis on primary care through DHS is based on the attributes of accessibility, continuity, comprehensiveness, coordination, and community participation [22]. The work situation leads to a high workload for the team due to the shortage of workforce, complexity of the population serves, and increased use of technology. Shared care and transition care through enhanced roles with supervision and skill transfer in the primary care team in DHS were established in the real situation. The result is in line with the type of skill-mix innovation in the establishment of teamwork and collaboration in multiprofessional teams of shared care, multiprofessional collaboration, and transitional care teams [61]. The type of skill-mix in DHS in Thailand was employed by delegation such as nurse transferring tasks to public health practitioner under supervision to achieve a better-quality care and integration of teamwork. Therefore, assessing the skill-mix role, designing courses, and monitoring outcomes are recommended to the policy maker.

The dimension of interprofessional reflection emerged in the work of the primary care team in Thailand. This scenario can be explained by the DHS’s emphasis on the appreciation and knowledge sharing of the team. Additionally, the National Health Security Office has provided voluntary training, called district health management learning (DHML), for the primary care team of 10–12 participants to learn together how to enhance collaborative practice since 2014 [6]. Consistent with a previous study, reflection enhances the outcomes of shared learning occasions and reflections on issues such as the role and importance of other professions, opportunities to learn with and from them, and their importance and generates a higher level of awareness that encompasses the broader context of patient care [62].

The strength of this study is that the IPC competencies instrument used a mixed method approach specific to the Thai DHS context. Nevertheless, this method can be replicable in other contexts, and these results can be documented in the body of knowledge on the IPC competencies of primary care teams. The application of this instrument is required to verify the validity and reliability of the instrument in real practice. The limitations are that the analysis is based on data from a single health region (Health region 2, which covers 5 provinces). However, the participants from this study used probability sampling in the quantitative phase. Future work will apply this instrument in other health regions. In addition, the internal validity of a test and ensures that the measurements obtained in one sitting are both representative and stable over time (i.e., test-retest reliability) is recommend for further studies to validate the instrument. While this instrument was designed as a self-evaluation, future work is required that uses objective evaluation indicators or other methods to ensure competencies. With the systematic development of this instrument, it can be used to assess the IPC competencies of primary care teams, and the data can contribute to tailor-made training programmes for primary care teams. An English version of the 49 items should be provided to advance research and practice of IPC.

## Conclusions

The IPC competencies instrument was confirmed the validity and reliability to assess the interprofessional competency of primary care teams. This will contribute to be the evidence on improving of IPC competencies in DHS.

## Data Availability

The data presented in this study are not publicly available due to the data are protected under the terms of the Naresuan University Ethical Committee for dissemination. However, available upon request from the corresponding author with the permission of the Naresuan University Ethical Committee.

## Abbreviations

IPC: Interprofessional Collaboration
WHO: World Health Organization
DHS: District Health System
ICCAS: Inter-professional Collaborative Competency Attainment Survey
CICS29: The Chiba Inter-professional Competency Scale
COPAN scale: The Collaboration Scale between Community Nurses (CNs) and General Practitioners (GPs) in Primary ﻿Health Care Teams
CPAT: The Collaborative Practice Assessment Tool
FGD: Focus Group Discussion
I-CVI: Item-level Content Validity Index
S-CVI/UA: Scale-level Content Validity Index through the Universal Agreement
EFA: Exploratory Factor Analysis
CFA: Confirmatory Factor Analysis
χ2/df: ﻿Chi-square per degree of freedom
CFI: Comparative Fit Index
RMSEA: Root Mean Square Error of Approximation
GFI: Goodness of Fit Index
SRMR: Standardized Root Mean Squared Residual
KMO: Kaiser-Meyer-Olkin
DHML: District Health Management Learning
